# Combination Therapy Comprising Paclitaxel and 5-Fluorouracil by Using Folic Acid Functionalized Bovine Milk Exosomes Improves the Therapeutic Efficacy against Breast Cancer

**DOI:** 10.3390/life12081143

**Published:** 2022-07-28

**Authors:** Dulla Naveen Kumar, Aiswarya Chaudhuri, Deepa Dehari, Anusmita Shekher, Subash C. Gupta, Shreyasi Majumdar, Sairam Krishnamurthy, Sanjay Singh, Dinesh Kumar, Ashish Kumar Agrawal

**Affiliations:** 1 Department of Pharmaceutical Engineering and Technology, Indian Institute of Technology (Banaras Hindu University), Varanasi 221005, India; dullanaveenkr.rs.phe20@itbhu.ac.in (D.N.K.); aiswaryachaudhuri.rs.phe20@itbhu.ac.in (A.C.); deepa.dehari.rs.phe18@itbhu.ac.in (D.D.); shreyasimajumdar.rs.phe18@itbhu.ac.im (S.M.); ksairam.phe@iitbhu.ac.in (S.K.); ssingh.phe@itbhu.ac.in (S.S.); dinesh.phe@itbhu.ac.in (D.K.); 2 Department of Biochemistry, Institute of Science, Banaras Hindu University, Varanasi 221005, India; anusmita.mhg.bhu15@gmail.com (A.S.); sgupta@bhu.ac.in (S.C.G.); 3 Department of General Surgery, Institute of Medical Science, Banaras Hindu University, Varanasi 221005, India; 4 Department of Biochemistry, All India Institute of Medical Science, Guwahati 781030, India; 5 Babasaheb Bhimrao Ambedkar University, Lucknow 226025, India

**Keywords:** exosomes, breast cancer, paclitaxel, 5-fluorouracil, co-delivery, chemotherapy

## Abstract

Paclitaxel (PAC) has been approved by FDA for clinical use (Taxol^®^), yet dose-dependent severe toxicity due to the adjuvant Cremophor EL^®^ in combination with ethanol is a major drawback. The drawbacks of the current therapy can be overcome by (i) finding a suitable vehicle that cannot only bypass the above adjuvant but also be used to deliver drugs orally and (ii) combining the PAC with some other chemotherapeutics to have the enhanced therapeutic efficacy. In the current work, we have used folic acid (FA) functionalized bovine milk-derived exosomes for oral delivery of PAC in combination with 5-fluorouracil (5-FU). Exosomes before and after the drug loading were found to have a particle size in the range of 80–100 nm, polydispersity index (PDI ~0.20), zeta potential (~−25 mV), entrapment efficiency (~82%), practical drug loading (~28%) and sustained drug release for 48 h. Significant decreases in IC_50_ were observed in the case of exosomes loaded drugs which further improved following the FA functionalization. FA functionalized coumarin-6-loaded exosomes showed remarkably higher cellular uptake in comparison with free coumarin-6. Moreover, FA-functionalized drug-loaded exosomes showed a higher apoptotic index with better control over cell migration. Collectively, data suggested the enhanced efficacy of the combination following its loading to the folic acid functionalized exosomes against breast cancer.

## 1. Introduction

Breast cancer remains the second leading cause of cancer-related deaths among women worldwide. In 2020, there were an estimated 2,261,419 new cases, which covers 11.7% of all types of cancers, and 684,996 deaths, thus comprising 6.9% of all types of deaths caused by cancer worldwide [1]. Radiation therapy, surgery, and chemotherapy are the therapy options available for breast cancer treatment. Among the stated therapeutic options, systemic chemotherapy is considered an attainable, potent, and cornerstone therapeutic strategy for the treatment of breast cancer [2,3].

In recent times, compared to monotherapy, combination therapy with multiple drugs has been raised as an effective approach in cancer therapy, as they exhibit synergistic activity by acting at different phases of the cell cycle or mechanistic pathway simultaneously as well as decrease the occurrence of multidrug resistance, resulting in an effective and synergistic anti-cancer activity [4]. In addition to synergism, combination therapy also decreases the dose-related toxicity associated with the single drug by improving their therapeutic efficiency via regulating their multi-facet pharmacodynamics [5]. Among various chemotherapeutics employed for the treatment of breast cancer, Paclitaxel (PAC) and 5-Fluorouracil (5-FU) are well-known first-line treatments for advanced metastatic breast cancer and non-small cell lung cancer [6].

Although PAC is considered one of the widely used anticancer drugs against breast cancer, its clinical application has been compromised due to its poor water solubility [7]. Although such a loophole has been covered by the application of a 50:50 mixture of Cremophor EL^®^ and ethanol with PAC, this combination exhibits serious dose-related toxicities such as hyperlipidemia, hypersensitivity reactions (anaphylaxis), aggregation of erythrocytes, abnormal lipoprotein patterns, prolonged and irreversible sensory neuropathy leading to demyelination and axonal degeneration [8]. To overcome these problems, Cremophor-free nanotechnology-based formulation, named Abraxane^®^ was approved by the US Food and Drug Administration for the treatment of breast cancer patients which is an albumin-bound nano-vector of PAC [9]. Although approval of Abraxane^®^ has shown lots of advantages over CrEL–PAC formulation, it exhibits certain severe adverse effects such as decreases in neutrophils and platelets, numbness, tingling, pain, or weakness in the hands or feet, and allergic (hypersensitivity) reactions [10]. Moreover, Abraxane^®^ is considered non-patient-friendly because its administration needs medical assistance and observation all the time [11].

The pyrimidine analog 5-FU is also the most potent cytotoxic agent, used to treat multiple solid tumors such as colorectal, breast, ovarian, and pancreatic cancer [12,13]. Moreover, 5-FU inhibits the growth and proliferation of solid tumors by inhibiting the thymidylate synthase and blocking the conversion of deoxyuridylic acid to deoxythymidylic acid, which further steers to apoptosis in cancer cells [14]. However, despite its effective anticancer activity against solid tumors, only 3% of the administered dose of 5-FU reaches the tumor site as it undergoes extensive metabolism via dihydropyridine dehydrogenase, thereby limiting its therapeutic efficiency [15]. Therefore, to maintain a high concentration at the acting site, continuous administration of the high dose is required, which further triggers non-selective bio-distribution of the drug leading to serious toxic effects, such as cardiac toxicity, hypovolemia, constipation, and azotemia [16]. To prevent cardiac toxicity, oral capecitabine was developed, which is a pro-drug of 5-FU that still, exhibits cardiac toxicity [17]. Several attempts with limited advantages have been made by scientists to deliver PAC and 5-FU in combinations to have an effective breast cancer therapy [18]. However, such combinatorial therapy still showed their individual adverse effects on the delivery system [19,20]. Therefore, a much-needed new delivery strategy becomes indispensable, which can reduce their off-site tissue toxicity, improve their therapeutic efficacy, could be cost reliable, have good bioavailability, and be biocompatible. In such a context, surface functionalized exosomes appeared as a boon in achieving the above-stated characteristics as well as providing a targeted delivery system, that may overcome most of the problems associated with the current treatment therapy [21,22].

Exosomes derived from bovine milk were used for the oral delivery of PAC and 5-FU; exosomes are considered an emerging drug-delivery technology described in recent times [23,24]. Exosomes are the biological nanoparticles present in all biological fluids, whose sizes range from 30 to 150 nm and have pulled an impressive consideration as a drug delivery system [25,26]. A study by our group recently showed that milk exosomes are biocompatible, able to cross the cellular barriers, and escape from the reticuloendothelial system, showing good oral bioavailability of the loaded drug and offering a cost-effective production of exosomes [27]. Such features could overcome all the limitations that the current delivery system encounters and could open a new platform for oral drug delivery of chemotherapeutics. Previously exosomal formulation had been successfully explored by our group for improving the oral delivery and reducing the toxicity of many anti-cancer drugs *viz* PAC, curcumin, cisplatin, and even siRNA [28,29,30,31,32,33]. Encouraged by our previous findings, we sought to extend the delivery of PAC and 5-FU combination through folic acid functionalized exosomes to achieve targeted delivery. 

In the present study, we endeavor to deliver PAC and 5-FU by exosomes functionalized with folic acid for targeted delivery. Exosomes were isolated from bovine milk and drug-loaded exosomes were prepared and characterized in terms of size, morphology, entrapment efficiency, powder X-ray diffraction, lyophilization, and in-vitro drug release. Furthermore, the efficacy of the combination and their formulations was further determined by in-vitro efficacy studies such as cytotoxicity, qualitative cellular uptake, quantitative cellular uptake, nuclear colocalization, intracellular trafficking, wound healing assay, and transwell migration assay of drug-loaded exosomes in MCF-7 and MDA-MB-231 cells.

## 2. Materials and Methods

### 2.1. Materials

Paclitaxel and 5-Fluorouracil were kindly gifted from Neon laboratories, Mumbai, Maharashtra, India, and Getwell pharma, Gurgaon, Haryana, India, respectively. LysoTracker^TM^ Red, DND-99 3-(4,5-dimethylthiazol-2-yl)-2,5-diphenyltetrazolium bromide (MTT), and fetal bovine serum (FBS) were purchased from Thermo Scientific, Waltham, MA, USA. Dulbecco’s Modified Eagle Medium (DMEM), anti-biotic and anti-mitotic solution, 4′,6-diamidino-2-phenylindole (DAPI), and trypsin solution were purchased from Himedia, Mumbai, India. Methanol, acetonitrile, and tween 80 were purchased from Merck, India. Coumarin-6, 1-Ethyl-3-(3-dimethyl aminopropyl)-carbodiimide (EDC), N hydroxyl succinimide (NHS), Bradford reagent, trehalose, and annexin v-cy3™ apoptosis detection kit was purchased from Sigma Aldrich, St Loius, MO, USA.

### 2.2. Isolation of Exosomes

The isolation of exosomes was performed from raw bovine milk that was collected from the dairy farm located inside the BHU campus, followed by a sequential centrifugation process as described previously [34], with slight modifications. Briefly, the raw milk was subjected to centrifugation at 13,000× *g* for 30 min using a Remi C-24 BL centrifuge (R-244 rotor), after which the supernatant obtained was ultracentrifuged for 1 h at 90,000× *g* (type 70 Ti rotor, Optima XPN-100, Beckman, Brea, CA, USA) to remove the microvesicles. The resultant supernatant was further separated and ultra-centrifuged for 2 h at 180,000× *g* (type 70 Ti rotor, Optima XPN-100, Beckman, USA), followed by the collection of pellets and their redispersion in PBS by using a hand homogenizer. The exosomal solution was then passed through 0.22 syringe filters to obtain uniform exosomes and until further use, they were stored at −80 °C. The protein content was estimated via the Bradford method.

### 2.3. Fabrication of Drug-Loaded Exosomes and Smart Exosomes

Drug-loaded exosomes were fabricated as discussed in [35]. Briefly, PAC and 5-FU were dissolved in methanol and PBS, respectively. These respective solutions of drugs were then mixed with respective exosomes, suspended in PBS solution at 1:9 ratios where 1 part is the drugs, while 9 part is the exosomes, followed by sonication (20% amplitude, 6 cycles 30 s for each cycle with two minutes gap between the cycles). After sonication, the exosomes were kept on ice for 60 min followed by centrifugation (10 min at 10,000× *g*) to remove the unbound drug and then ultracentrifugation at 190,000 × *g* for 2 h to isolate the drug-loaded exosomes. Exosomal pellets were redispersed in PBS and further surface functionalized by using activated folic acid. Folic acid was activated by using click chemistry as per our previous protocol [36]. Briefly, folic acid, EDC, and NHS were dissolved in dried DMSO at the ratio of 1:1:1.2 followed by stirring overnight at ambient temperature [37,38,39]. The so-formed activated folic acid was then separated by precipitating it into chilled diethyl ether. Activated folic acid was then mixed and incubated with exosomes at 4 °C for 4 h. Folic acid-functionalized drug-loaded exosomes (FA-Exo-PAC and FA-Exo-5-FU) were isolated by following the same protocol as above. 

### 2.4. Particle Size, Polydispersity Index, Zeta Potential, and Entrapment Efficiency

The particle size, polydispersity index (PDI), and zeta potential of Exo, Exo-5-FU, Exo-PAC, FA-Exo-PAC, and FA-Exo-5-FU were measured by the dynamic light scattering (DLS) method [40]. Briefly, small aliquots (100 μL) of different formulations were diluted with 1.4 mL of cold PBS, filtered through a 0.22 μm PVDF syringe filter, and measured for particle size, PDI, and zeta potential via Zeta-sizer (Delsa^TM^Nano, Beckman coulter, Brea, CA, USA). 

To evaluate the entrapment efficiency and drug loading capacity of the exosomes, 100 μL of drug-loaded exosomes was mixed with 900 μL methanol to extract drugs from exosomes, followed by 10-min centrifugation at 10,000× *g* for the separation of exosomal precipitated proteins. The supernatant was suitably diluted with PBS, and PAC and 5-FU concentrations were analyzed by UV-spectrophotometer. The percent of entrapment efficiency (%EE) and drug loading were calculated by applying the formula depicted below [41,42].

$$\mathrm{EE}\%=\;\frac{\left(W_{\mathrm{total}\;\mathrm{drug}}-W_{\mathrm{free}\;\mathrm{drug}}\right)}{\left(W_{\mathrm{total}\;\mathrm{drug}}\right)}\times 100$$

$$\mathrm{Drug}\;\mathrm{loading}=\frac{\left(W_{\mathrm{total}\;\mathrm{drug}}-W_{\mathrm{free}\;\mathrm{drug}}\right)}{\left(\mathrm{total}\;\mathrm{weight}\right)*}\times 100$$

* Total weight = weight of the exosomes and weight of the total

### 2.5. Scanning Electron Microscopy (SEM) for Morphology Analysis

Exosomes and drug-loaded exosomes (30 μL) were diluted to 500-folds using PBS and filtered through a 0.22 μm PVDF syringe filter [43]. Filtered exosomal dispersions (10 μL) were added onto a clean glass slide and air-dried for 1 h, after which the slides were coated with gold, and images were taken using Nova Nano SEM 450 (FEI, Hillsboro, OR, USA) at 15.0 KV [44]. 

### 2.6. In-Vitro Drug Release Study

In-vitro drug release of different formulations was assessed using the dialysis bag method [45] where PBS (0.1% of Tween 80 was added in PBS to maintain the sink condition) was employed as the dissolution medium. Before the study, the dialysis bags were activated by soaking in PBS overnight as per the manufacturer’s protocol. Briefly, a known amount of drug-loaded exosomes/drug solutions (equivalent to 1 mg of drug) were filled in dialysis bags (MWCO, 10 kDa, Himedia) and immersed in 50 mL of release media (PBS with 0.1% Tween 80) in a separate set of experimentations [46,47]. Aliquots (1 mL) were withdrawn at different time intervals (0, 1, 2, 4, 8, 12, 24, and 48 h) and replaced with equal amounts of fresh media [48]. The % cumulative release of 5-FU and PAC were analyzed by the developed method as discussed in Section 2.4.

### 2.7. X-ray Diffraction Analysis

The X-ray diffraction pattern of PAC, 5-FU, exosomes, Exo-PAC, and Exo-5-FU were analyzed using Rigaku SmartLab 9kW Powder type (Rigaku Corporation, Sendagaya, Shibuya-ku, Japan) with diffraction angle (2θ) ranging from 5 to 40° and scan rate of 5°/min [49,50,51]. The powdered samples were dispersed in water and then placed on the sample holder, which was then placed on a goniometer to record the diffraction pattern using the DTEX detector [20].

### 2.8. Freeze Drying

Exosomes and drug-loaded exosomes were freeze-dried as discussed in [52] with slight modifications. Cryoprotectant (Trehalose 5% *w/v*) was added to the exosomal dispersions and placed in a deep freezer for 24 h (Thermo fisher). The samples were then lyophilized for 12 h using Labocon LFD-BT-101 (Labocon scientific Ltd. Fleet, Hampshire, UK) and stored in a cool place until use. Before use, the freeze-dried exosomes were rehydrated with PBS and vortexed immediately.

### 2.9. Cell Culture and Conditions

Breast cancer cell lines (MCF-7 and MDA-MB-231) were procured from the National Center for Cell Science (NCCS), Pune, India, cultured in DMEM (Dulbecco’s modified eagle’s medium), supplemented with 10% FBS (Gibco, Thermo scientific, Waltham, MA, USA) and 1% anti-biotic anti-mitotic solution (Himedia, Mumbai, India).

### 2.10. In-Vitro Cytotoxicity

For the analysis of the cytotoxicity of drug-loaded exosomes, an MTT assay was performed [53]. Briefly, the cells were seeded at the density of 1 × 10^4^ cells/well followed by overnight incubation for the attachment of the cells. After 24 h of incubation, the media was aspirated, and fresh media was added comprising of Exo, PAC, 5-FU, PAC+5-FU, Exo-PAC, Exo-5-FU, Exo-PAC+Exo-5-FU, FA-Exo-PAC+FA-Exo-5-FU at different concentrations and incubated for 24, 48, and 72 h (we were taken exosomes concentrations as µg/mL, and the concentration exosomes were measured by Bradford protein estimation assay, the amount of protein concentration is directly proportional to exosomes content). After the completion of treatment, media was aspirated, and fresh media (100 µL) containing 500 µg/mL of MTT was added to each well and incubated for 4 h. Following the incubation, 100 µL of DMSO was added to each well to dissolve the formazan crystals, and then the optical density was recorded at 570 nm using a Bio-Tek plate reader.

### 2.11. Qualitative Cell Uptake and Nuclear Co-Localization

Coumarin-6 (C-6) was used as a model dye to analyze the qualitative cellular uptake of exosomes [54]. Briefly, MDA-MB-231 cell lines were seeded in 8 chambered well plates (SPL life sciences) at the density of 5 × 10^5^ cells/well and incubated overnight with complete media. After incubating for 24 h, the media was aspirated, and fresh media was added comprising of C-6, C-6 loaded exosomes, and FA-C-6 loaded exosomes followed by incubation for 4 h. After 4 h incubation, the cells were washed with chilled PBS and were permeabilized with ethanol for 30 min. The nuclei of MDA-MB-231 cells were further stained with DAPI (10 µg/mL) (Himedia, Mumbai, India) and observed under a fluorescence microscope (EVOS FLoid).

### 2.12. Quantitative Cell Uptake Study

A quantitative cell uptake assay was performed as per our previous reports [55]. Briefly, 12 well plates were seeded with MCF-7 and MDA-MB-231 cells at the density of 5 × 10^5^ cells/well and incubated overnight with complete media. After incubation for 24 h, media was aspirated, and fresh media was added comprising of PAC, 5-FU, and their formulations (equivalent concentrations of 1 µM and 2 µM of PAC and 5-FU, respectively) and again incubated for different time intervals (0.5, 1, 2, 4 h). After different time intervals, media were aspirated and cells were washed with cold PBS to extrude the extracellular drugs, followed by treatment with methanol. Following this, the cells were harvested with trypsin and centrifuged for 10 min at 10,000 rpm. The supernatant obtained was then subjected to UV-spectroscopy (Agilent, Santa Clara, CA, USA) for the quantification of PAC and 5-FU.

### 2.13. Intracellular Trafficking Assay

The intracellular localization assay was performed by Lysotracker Red^®^ (Invitrogen, Carlsbad, CA, USA), which is a dye used to detect biodistribution into organelles such as lysosomes and endosomes [56]. Briefly, 12 well plates were seeded with cell lines (MCF-7 and MDA-MB-231) at the density of 5 × 10^4^ cells/well and incubated for 24 h. After incubation, cells were treated for 2 h with different exosomal formulations followed by washing with PBS. Fresh media was then added containing Lysotracker red dye (1 µM) and incubated for 30 min followed by washing with PBS and observing under a fluorescence microscope (Olympus) [57].

### 2.14. Annexin-V Apoptotic Assay

The in-vitro therapeutic potential of PAC and 5-FU loaded exosomes was further estimated by analyzing their capacity in inducing apoptosis in MCF-7 and MDA-MB-231 cells, via standard phosphatidylserine membrane externalization assay based on annexin-V binding [58]. Briefly, the cells were seeded at the density of 5 × 10^4^ cells/well in 8 well-chambered cell culture slides (Mat Tek, Asland, MA, USA) and incubated for 24 h at 37 °C and 5% CO_2_ for the attachment of the cells. Following the period of incubation, the media was aspirated, and cells were treated for 6 h with different PAC and 5-FU loaded exosomes (equivalent concentrations of 1 and 2 µM of PAC and 5-FU, respectively) [59]. After the treatment period, cells were washed three times with cold PBS and stained with Annexin-VCy3.18 conjugate (AnnCy3) and 6-carboxyfluorescein diacetate (6-CFDA) (Annexin V-Cy3TMApoptosis Detection Kit, Sigma, St Loius, MO, USA) for 10 min followed by observation under a fluorescence microscope (EVOS FLoid).

### 2.15. Wound Healing Assay

The wound-healing assay of developed exosomal formulations was performed as described in [60]. Briefly, 100 µL of both MCF-7 and MDA-MB-231 cells were seeded at the density of 1 × 10^5^ cells/mL in the Culture-insert 2 well (Ibidi, Lochhamer Schlag, Gräfelfing, Germany). The cells were then allowed to incubate for 24 h to attach the cells to the wells. Then, a wound was formed while removing the culture inserts gently with the help of sterile forceps. Then, cells were washed with pre-cooled PBS to discard the dead cells and cell debris, followed by the addition of fresh media containing different PAC and 5-FU, loaded exosomes (equivalent concentrations of 0.5 and 1 µM of PAC and 5-FU, respectively), and empty exosomes at 10 µg/mL and incubation for 24 h. The wounds were observed under the microscope (Dewinter, New Delhi, India) at 0, 8, and 24 h. At each time point, a microphotograph was taken, and the wound areas for each photograph were quantitatively determined using Image J software.

### 2.16. Cell Migration Assay

The cell migration assay was performed using Transwell chambers (Sigma Aldrich, USA) as described previously [61]. Briefly, both MCF-7 and MDA-MB-231 cells were pretreated with Exo, PAC, 5-FU, PAC+5-FU, Exo-PAC, Exo-5-FU, Exo-PAC+Exo-5-FU, and FA-Exo-PAC+FA-Exo-5-FU (equivalent concentrations of 0.5 and 1 µM of PAC and 5-FU, respectively) for 60 min, followed by harvesting of cells with trypsin and counting of cells. Then, 200 µL of cell suspension (4 × 10^2^ cells/100 µL) were added to all the top compartments of the transwell chambers and the complete DMEM media containing 10% of FBS were added to the bottom compartments of the transwell chamber which further act as a chemoattractant used to stimulate the migration of cancer cells from the top to the bottom compartment. The cells were then incubated for 24 h to allow cell migration. After 24 h of incubation, the migrated cells were fixed with 4% paraformaldehyde, permeabilized with methanol, and stained using 0.2% crystal violate. The number of migrated cells was counted in five random fields under an inverted microscope (Dewinter, New Delhi, India).

### 2.17. Statistical Analysis

All data are expressed as mean ± standard deviation (SD). The statistical analysis was performed using a student’s *t*-test in Graph Pad Prism Version 5.0 (GraphPad Software, Inc., San Diego, CA, USA). Image J software (National Institute of Health, Bethesda, MD, USA) was used for the analysis of the wound healing study and CompuSyn software was used for the combination studies.

## 3. Results and Discussion

### 3.1. Isolation and Characterization of Exosomes and Drug-Loaded Exosomes

**Figure 1:**
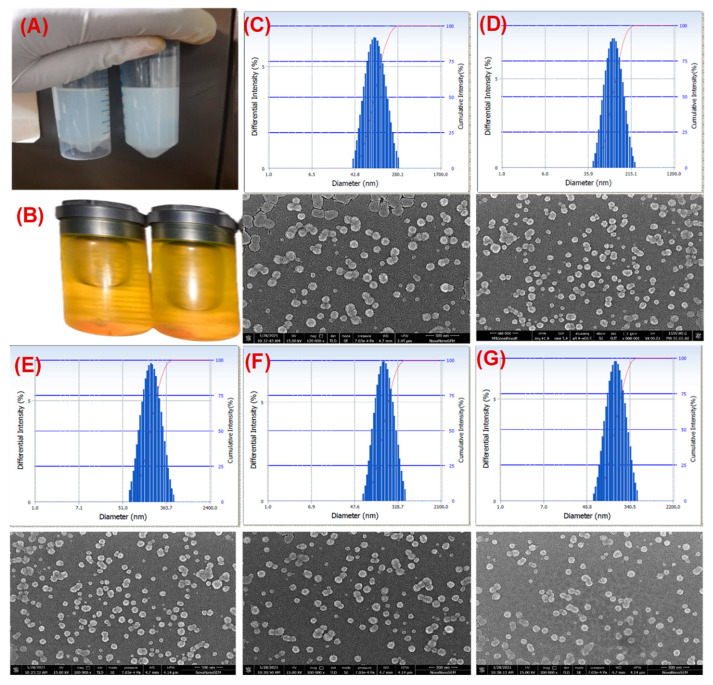
Preparation and characterization of drug-loaded exosomes. (**A**) represents the drug-loaded exosomes while (**B**) represents the FA acid-functionalized drug-loaded exosomes. (**C**–**G**) represents the particle size determination by zeta sizer and surface morphology determination by SEM for (**C**) exosomes (**D**) Exo-PAC (**E**) Exo-5-FU (**F**) FA-Exo-PAC, and (**G**) FA-Exo-5-FU.

Exosomes isolated from bovine milk showed an average particle size and PDI of 83.5 ± 2.6, and 0.12 ± 0.03, respectively, while the PAC-loaded exosomes (Exo-PAC) and 5-FU loaded exosomes (Exo-5-FU) showed an average particle size 86.6 ± 2.6 and 88.2 ± 2.8 nm while PDI of 0.137 ± 0.002, and 0.140 ± 0.004, respectively (Figure 1C–G, and Table 1). This data suggested an insignificant effect of drug loading on particle size and PDI. However, we observed a slight change in the zeta potential of Exo-PAC (−28.28 ± 1.8 mV), and Exo-5-FU (−27 ± 1.6 mV), as compared to plain exosomes (−23 ± 1.2 mV) (Figure 1C–G and Table 1). The practical drug loading was found to be 28 ± 3.9% in Exo-PAC and 19 ± 4.6% in Exo-5-FU, 26 ± 2.6 in FA-Exo-PAC, and 17 ± 2.9 in FA-Exo-5-FU while the entrapment efficiency was found to be 75–85% (Table 1). Surface morphology determination by scanning electron microscopy (SEM) disclosed that the exosomes are spherical (Figure 1C–G), which remained unchanged even after drug loading. However, the size of exosomes and drug-loaded exosomes is less in SEM analysis (exosomes 60–80 nm and drug-loaded exosomes 65–85 nm) as compared to the size observed with the particle size analyzer.

An insignificant increment in size was observed in drug-loaded exosomes (~88.2 ± 2.8) as compared to plain exosomes (~83.5 ± 2.6) which are in line with previous observations [62,63]. The size of the bovine milk exosomes as measured by the DLS technique (~84 nm) was found to be slightly bigger when observed via SEM (~65–85), which could be assigned to the obvious reason of measuring the hydrodynamic diameter in case of DLS rather than actual size measurement by SEM. No visible changes in the surface morphology of exosomes followed by the sonication method for loading drugs further demonstrated the compatibility of the loading method without compromising the morphology of exosomes. The surface potential of exosomes was measured by Zetasizer and it was found to be −23 mV which could be due to the carboxyl groups found over the surface of the exosomes. Further, the surface potential was shifted to ~28 in drug-loaded exosomes, which we could assume due to the interaction of PAC and 5-FU with the carboxyl groups on the surface of the exosomes.

### 3.2. Powder X-ray Diffraction

**Figure 2:**
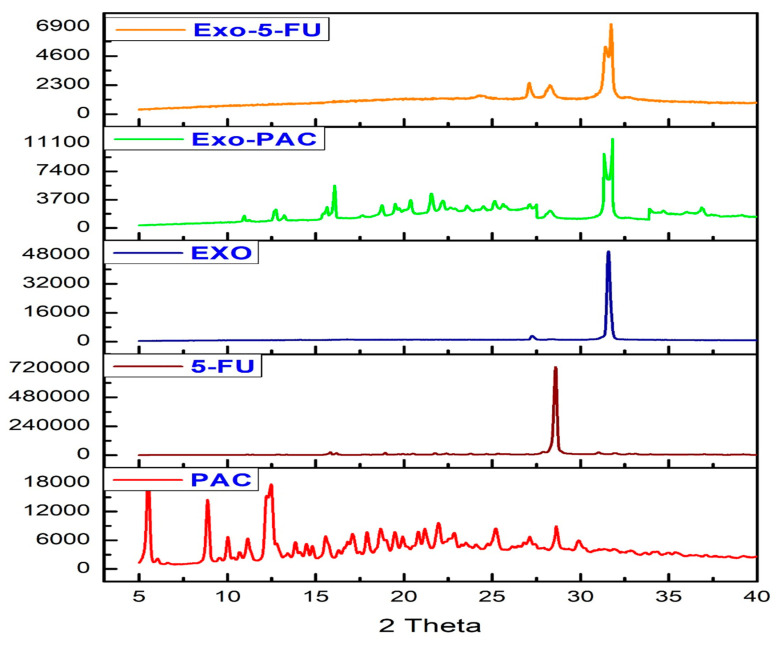
X-ray powder diffraction of Exosomes, PAC, 5-FU, Exo-PAC, and Exo-5-FU.

The diffraction pattern of PAC, 5-FU, Exosomes, Exo-PAC and Exo-5-FU was shown in Figure 2. Several peaks were observed at 6.2, 9.6, 10.1, 11.2, 12.5, 14.6, 18, 18.6, 22.5, 25.6, and 28.2 in the case of PAC while one sharp peak at 28.2 was observed for 5-FU. One sharp peak at 31.5 was observed in the case of Exo alone while Exo-PAC showed peaks at 12.5, 15.2, 22.6, 28.3, and 31.8 while Exo-5-FU showed peaks at 27.4, 28.2, and 31.6. The presence of peaks in the case of PAC and 5-FU indicated the crystalline nature of the compounds. Following the loading, some of the peaks disappeared while some were still present, although with lesser intensity. The lesser intensity could be explained by the encapsulation of the drug within the exosomes; however, the presence of some of the peaks could explain the presence of some amount of drug over the surface due to the hydrophobic interaction between the exosomal surface proteins and the drug molecules. Overall, the results of PXRD suggested that a major part of the drugs is encapsulated within the exosomes while a part is also present over the exosomal surface.

### 3.3. Freeze Drying

Freeze drying is a technique in which the water molecules are removed from the formulations to prevent aggregation and provide physical stability to the pharmaceutical product. In the study, Trehalose (5%) was used as a cryoprotectant for the freeze-drying of exosomes. It was observed that lyophilization of Folic acid-functionalized drug-loaded exosomes with the trehalose prevented the aggregation of particles, which was found to be following the previous observations by others [64]. After redispersing the lyophilized exosomes in PBS, the size and the drug content of the exosomes remained unchanged (Table 2).

### 3.4. In-Vitro Drug Release

**Figure 3:**
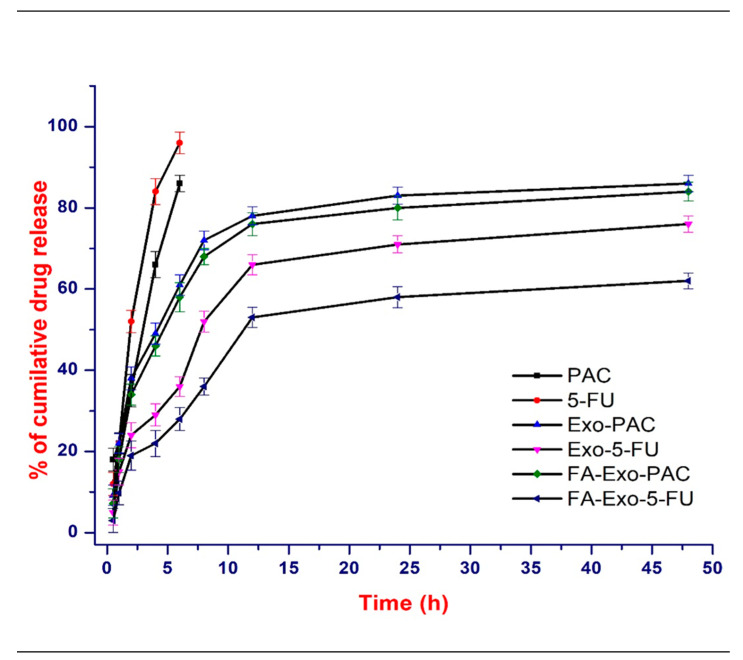
In-vitro drug release profile of PAC, 5-FU, Exo-PAC, Exo-5-FU, FA-Exo-PAC, and FA-Exo-5-FU in PBS (pH 7.4) containing 0.05% of tween 80 at 37 °C (*n* = 3).

The in-vitro release of PAC and 5-FU from exosomes was investigated in PBS (pH 7.4) at 37 °C and a biphasic release pattern was observed in the case of both the drugs (Figure 3). A burst release was observed in the first 1 h, with almost 20% release of 5-FU and 25% release of PAC followed by a sustained release for up to 48 h. However, in the case of free drugs, an almost 100% release was observed within 8 h. The burst release may be accounted for the surface adsorbed drug attached via hydrophobic interaction while the sustained release may be accounted for the diffusion of the PAC and 5-FU entrapped within the exosomes. This data may further be explained by our PXRD observations, where part of the drug was present over the surface while the rest was entrapped within the exosomes. It was suggested that the biphasic release pattern of drugs from the exosomes can be used to inhibit cancer growth in both the short and long run, as the burst release can be used as an immediate measure while the sustained drug release can be used as a prolonged and continuous measure against cancer cells.

### 3.5. Cytotoxicity Study

**Figure 4:**
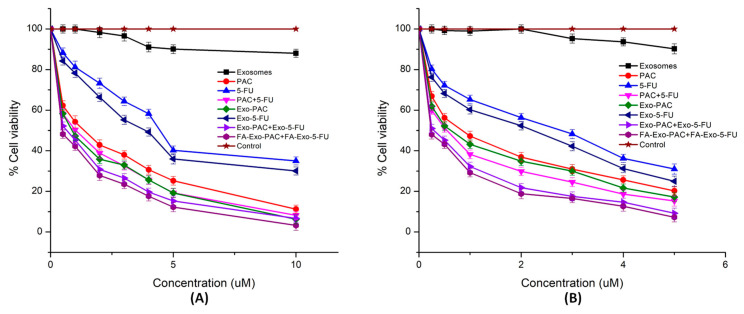
In-vitro cytotoxic results performed by MTT assay in (**A**) MCF-7 cell lines; (**B**) MDA-MB-231 cell lines following the different treatments up to 24 h.

An MTT assay was employed for the analysis of the anticancer efficiency of the developed exosomal formulations against breast cancer cells (MCF-7 and MDA-MB-231). The MTT assay was performed by incubating different treatments at different concentrations for 24, 48, and 72 h (Table 3). The IC_50_ values of Exo, PAC, 5-FU, PAC+5-FU, Exo-PAC, Exo-5-FU, and Exo-PAC+Exo-5-FU were found to be 13 ± 1.6, 0.6 ± 0.5, 2.9 ± 0.5, 0.52 ± 0.11, 0.54 ± 0.1, 2.2 ± 0.4, and 0.28 ± 0.12 μM, respectively, after 48 h treatment in MCF-7 cells. Furthermore, the IC_50_ value was found to be decreased to 0.11 ± 0.08 μM in the case of FA functionalized exosomes. As the incubation period was increased to 72 h, the IC_50_ values of Exo, PAC, 5-FU, PAC+5-FU, Exo-PAC, Exo-5-FU, Exo-PAC+Exo-5-FU, and FA-Exo-PAC+FA-Exo-5-FU were found to be 15.12 ± 1.4, 0.78 ± 0.2, 3.6 ± 0.3, 0.69 ± 0.21, 0.67 ± 0.1, 2.9 ± 0.31, 0.48 ± 0.2 and 0.18 ± 0.09 μM. Similar results were observed in MDA-MB-231 cells (Figure 4 and Table 3). Interestingly, plain exosomes at the concentration of 15 μg/mL also showed inhibitory action on both the cell lines. The inhibitory effect of plain exosomes might be due to the presence of several proteins and nucleic acids including miRNA that may act as tumor suppressor agents. The cytotoxicity results further indicated that the PAC and 5-FU loaded exosomal formulation showed increased cytotoxicity as compared to free-PAC and 5-FU, which might be because of the effective internalization of exosomes into cancer cells. Moreover, FA functionalized exosomes had lowered IC_50_ values which might be due to the strong interaction of FA with folate receptors and thus the enhanced cellular uptake. However, further investigation may be required to identify the percentage of FA functionalization that shows the highest cytotoxicity effects.

### 3.6. Cellular Uptake and Nuclear Colocalization

**Figure 5:**
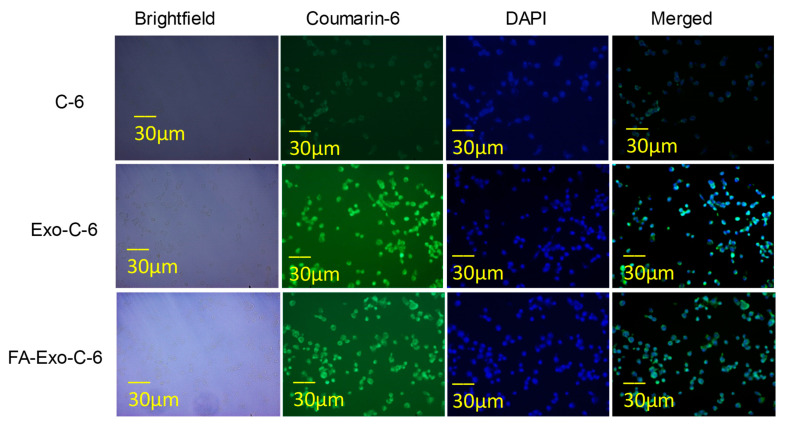
Qualitative cellular uptake in MDA-MB-231 cell lines; free Coumarin-6, Exo-coumarin-6, FA-Exo-C-6. Following the treatment for 4 h, the cells were permeabilized with ethanol and stained with DAPI. Cells were observed in Brightfield, the green channel for C-6, and the blue channel for DAPI. The green channel and blue channel were further merged to see the colocalization within the cells.

To reinforce our assumption of enhanced cellular uptake owing to the conjugation of FA to the exosomes, a cellular uptake study was assessed in MDA-MB-231 cell lines. The selection of the MDA-MB-231 for this study was based upon the previous reports in which this cell line has been reported to have 1.76 times more expression of FA as compared to MCF-7 [65]. As shown in Figure 5, the green, fluorescent panel indicated that the FA conjugated C-6 loaded Exo- showed a relatively enhanced cellular uptake in MDA-MB-231 cell lines, which revealed improved internalization of exosomes, compared to free C-6. Additionally, horizontal and vertical line analysis was performed that revealed significant superimposing of green fluorescence of C-6 with the vibrations, while lining vibrations are due to the presence of cells. Moreover, to confirm the presence of exosomes inside the cell, the cells have been stained with DAPI, and it was observed that the exosomal particles are present inside the cell rather than outside the cell.

### 3.7. Quantitative Cell Uptake Assay

**Figure 6:**
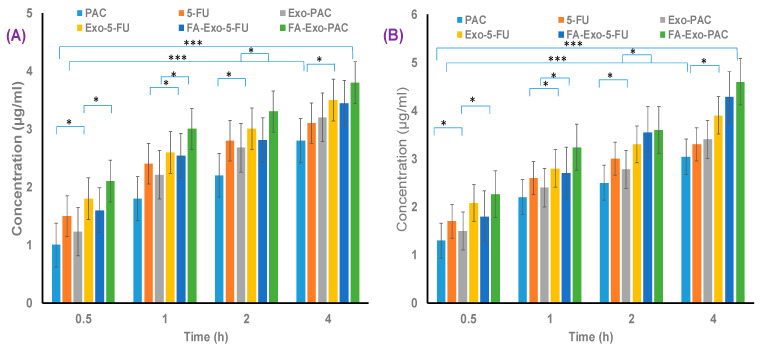
Quantitative uptake measurement of, PAC, 5-FU, PAC+5-FU, Exo-PAC, Exo-5-FU, FA-Exo-PAC, and FA-Exo-5-FU after 0.5, 1, 2, 4 h incubation period (**A**) In MCF-cell lines, (**B**) in MDA-MB-231 cell lines (* Significance difference at *p* ˂ 0.05, *** significance difference at *p* ˂ 0.001).

Time-dependent quantitative uptake of PAC, 5-FU and drug-loaded exosomal formulations were performed in MCF-7 and MDA-MB-231 cell lines. As shown in Figure 6, significant quantitative cellular uptake of PAC and 5-FU were observed in the FA functionalized drug-loaded exosomes as compared to free drug and non-functionalized drug-loaded exosomes.

The increased cellular uptake of FA functionalized exosomes was due to the strong binding of folic acid attached to the exosomes to the folate receptors overexpressed on the breast cancer cells followed by their internalization into the cancer cells. These findings further support the superiority of the functionalized system over the non-functionalized one.

### 3.8. Intracellular Trafficking of Free Drugs and Exosomal Formulations

**Figure 7:**
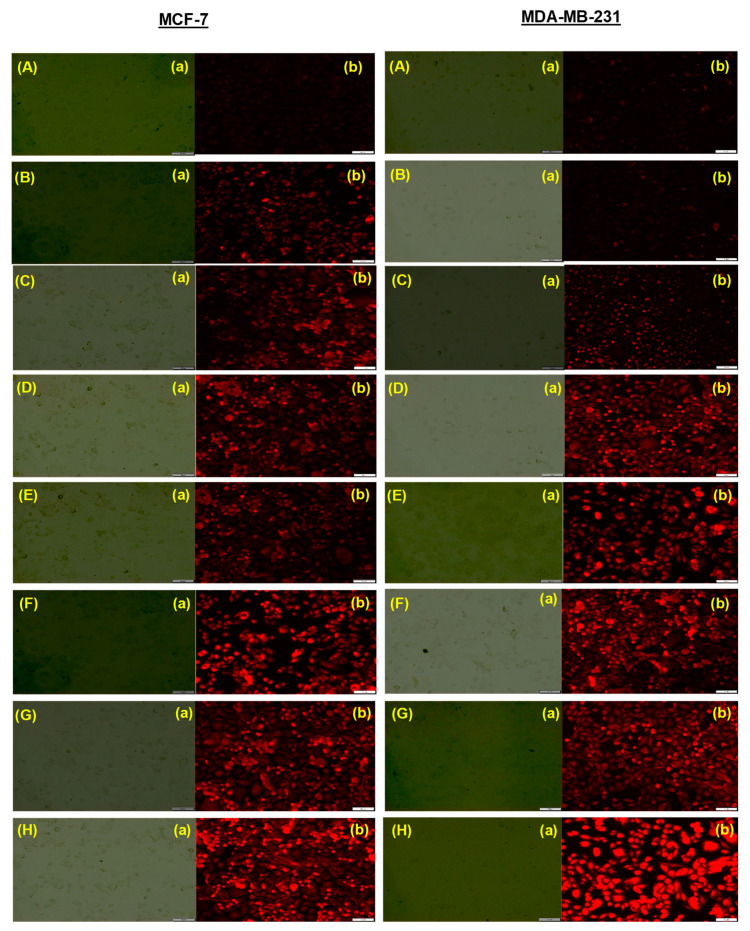
Intracellular fate of (**A**) Exo, (**B**) PAC, (**C**) 5-FU, (**D**) PAC+5-FU, (**E**) Exo-PAC, (**F**) Exo-5-FU, (**G**) Exo-PAC+Exo-5-FU, and (**H**) FA-Exo-PAC+FA-Exo-5-FU as shown by staining with Lyso Tracker^®^ Red DND-99 after 24 h’ treatment. Panel (**a**) Images under Brightfield microscope, (**b**) Images under red fluorescence.

The subcellular transport of Exo, PAC, 5-FU, the combination of PAC and 5-FU, Exo-PAC, Exo-5-FU, Exo-PAC+Exo-5-FU, and FA-Exo-PAC+FA-Exo-5-FU were assessed using LysoTracker^®^ Red dye. It was observed that a significant number of lysosomes were formed in MCF-7 and MBA-MD-231 cells following the treatment with Exo, PAC, 5-FU, PAC+5-FU, Exo-PAC, Exo-5-FU, Exo-PAC+Exo-5-FU, and FA-Exo-PAC+FA-Exo-5-FU following the treatment for 24 h (Figure 7). Moreover, FA-Exo-PAC+FA-Exo-5-FU treated cells exhibited enhanced red fluorescence intensity as shown in Figure 7, as compared to the cells treated with free drugs, their combination, and control (without treatment). Furthermore, an evaluation of vertical and horizontal line series was conducted, which exhibited relevant superimposing of red fluorescence signals with the white lines of vibrations (corresponding with cells signals) within the differential interface contrast images of cells (Figure 7). Additionally, LysoTracker^®^ red fluorescence also superimposes with the green fluorescence revealing the existence of FA-Exo-PAC+FA-Exo-5-FU within the peripheral region of MCF-7 and MDA-MB-231 cells, which further confirmed that the FA-Exo-PAC+FA-Exo-5-FU is localized within the lysosomes.

### 3.9. Annexin-V Apoptosis Assay

**Figure 8:**
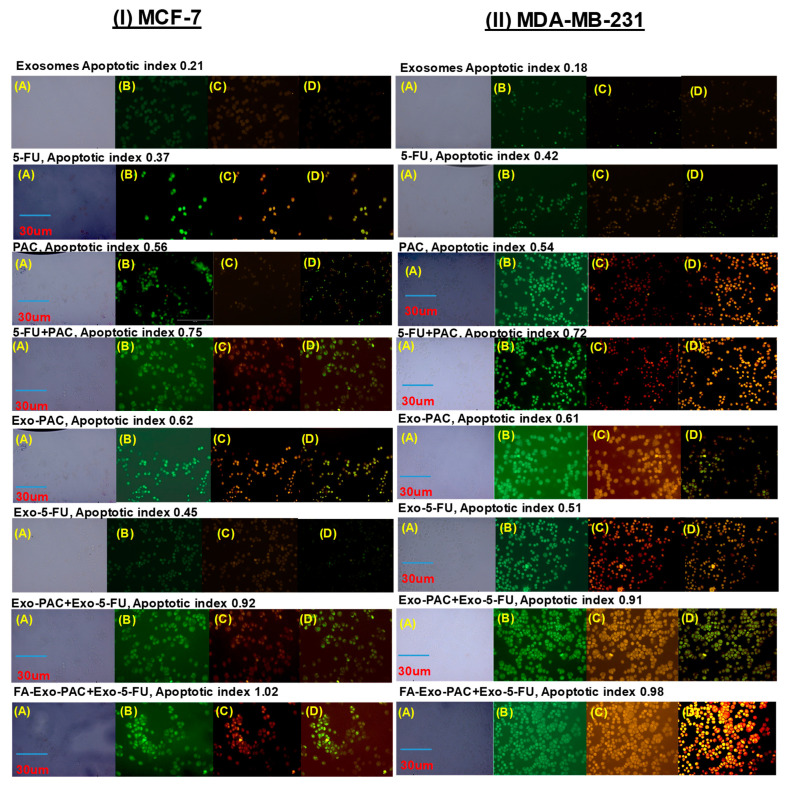
Apoptosis assay of free drugs and drugs loaded exosomes against (**I**) MCF-7 and (**II**) MDA-MB-231 cells; (**A**) brightfield, (**B**) green fluorescence due to staining with 6-carboxyfluorescein (indicate live cells), (**C**) red fluorescence due to staining with Annexin Cy3.18 (indicate dead cells), (**D**) both green and red fluorescence indicates early apoptosis incubation of 6 h.

Most of the chemotherapeutics inhibit cellular growth by inducing apoptosis, which is also considered one of the major mechanisms of action for most of the anti-cancer drugs. It has been reported that both the PAC and 5-FU inhibit the cell growth through the apoptosis; hence. to prove the establishment, an annexin-V apoptosis assay was performed in MCF-7 and MDA-MB-231 cells. Drug-loaded exosomes showed an increased apoptosis index in both MCF-7 and MDA-MB-231 cell lines as compared to free drugs and their combination. Figure 8 shows that exosomes treated cells indicated higher red fluorescence from Annexin Cy3.18 conjugate (cell apoptosis marker dye, panel b), as compared to free drug and their combination (green indicate live cells, red indicates dead cells, and cells in both colors indicate early apoptosis). On further evaluation, it was found that in the MCF-7 and MDA-MB-231 cell lines, the combination of PAC and 5-FU showed an apoptotic index of 0.92 and 0.91, respectively, while the FA-Exo-PAC+FA-Exo-5-FU exhibited an increased apoptotic index of 1.02 and 0.98, respectively. Hence, from the study, it was observed that PAC and 5-FU loaded exosomes showed increased apoptosis as compared to free drugs as well their combination. Moreover, the results obtained in the apoptosis study were found to be in line with our earlier observation during the cytotoxicity study.

### 3.10. Wound Healing and Migration Assay

**Figure 9:**
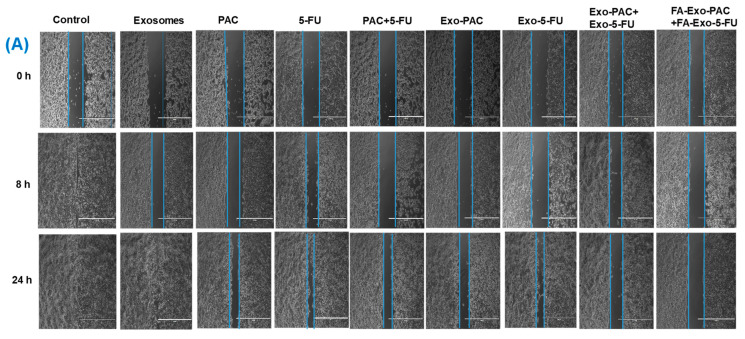

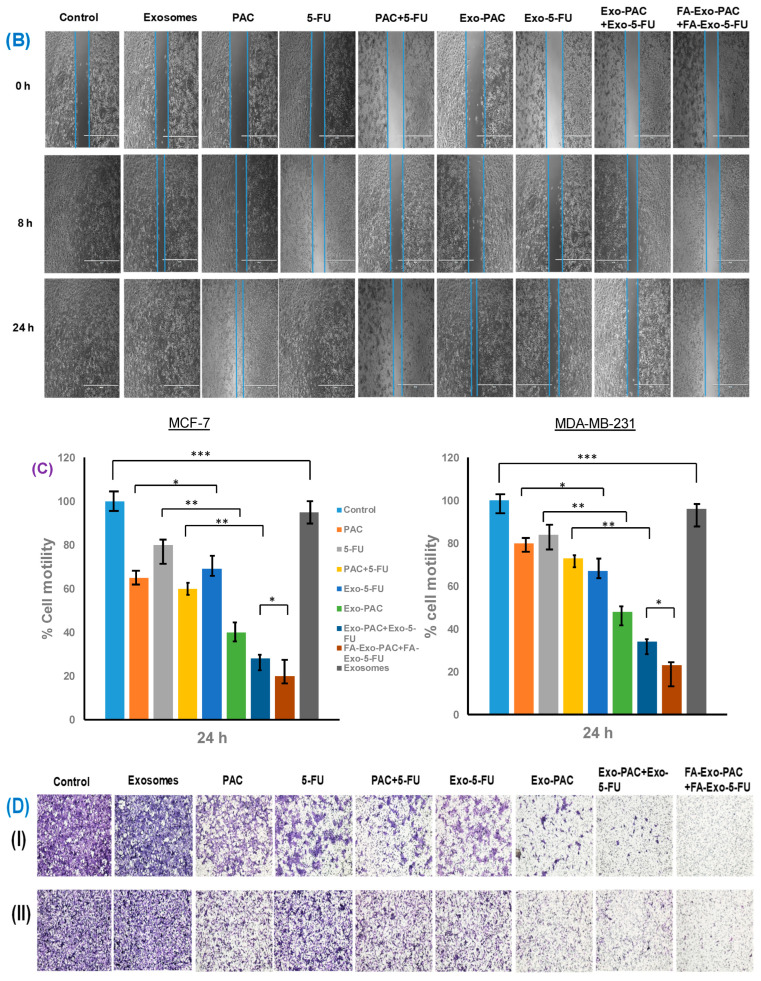
Wound healing and migration assay. Wound healing assay showing inhibitory effects of different treatments in (**A**) MCF-7 cell lines, (**B**) MDA-MB-231 cell lines, (**C**) percentage inhibition of cell motility, and (**D**) cell migration assay ((**I**) MCF-7 cells, and (**II**) MDA-MB-231 cella). Values are represented as mean ± SD, * *p* ˂ 0.05, ** *p* ˂ 0.01, *** *p* ˂ 0.001).

Increased migration ability of cancer cells is known to be demonstrative of higher metastatic capability in most solid tumors such as breast cancer. Therefore, to assess the anti-migratory property of PAC, 5-FU, and drugs-loaded exosomal formulations in MDA-MB-231 and MCF-7 cell lines, wound healing (scratch assay) and migration assays were performed. However, dissimilar from the cell cytotoxicity assay (Figure 4), in the wound healing and migration assays, the cells were incubated for 24 h at low concentrations of PAC and 5-FU loaded exosomes. For wound healing assay (Figure 9A,B), both the cell lines were pre-incubated with PAC and 5-FU-loaded exosomal formulations (PAC equivalent to 0.5 µM and 5-FU equivalent to 1 µM). From the results, it was observed that the FA-Exo-PAC+FA-Exo-5-FU has significantly prevented the migration of both MCF-7 and MDA-MB-231 cells. Moreover, it was observed that in the presence of PAC and 5-FU loaded exosomal formulations, only 40–50% of wound areas were covered in both the cell lines, indicating a significant reduction in migration of both the cell line at 24 h (* *p* ˂ 0.05). However, the control group (only media) showed more than 98% coverage of the wound areas (scratches) within 24 h in both the cell lines demonstrating higher cell migration. However, the combination of PAC and 5-FU loaded exosomes witnessed a significant reduction in cell mobility, which was only 20–30% cell migration. Interestingly, folic acid functionalized PAC and 5-FU loaded exosomal formulations had only 15–20% of cell migration in both cells, which revealed that the targeted delivery decreases cell migration compared to non-targeted delivery in breast cancer.

To further support the results of the wound healing study, a Transwell migration assay was performed. As shown in Figure 9, folic acid functionalized exosomal formulations showed a significant reduction in cell migration at a dose equivalent to 0.5 µM and 1 µM of PAC and 5-FU, respectively, as compared to non-functionalized exosomal formulation (* *p* ˂ 0.001). Images of migrated cells and the number of cells counted in 5 random areas are shown in Figure 9D, which supported the fact that the MCF-7 cell lines showed higher migration than MDA-MB-231.

## 4. Conclusions

In the current study, a combination of PAC and 5-FU was proposed by using bovine Milk exosomes as a delivery vehicle along with folic acid functionalization for target deliverability. Here, the exosomes were isolated from raw bovine milk via sequential centrifugation followed by loading of drugs (PAC and 5-FU). It was observed that PAC and 5-FU loaded exosomes exhibited the particle size from 80–100 nm, PDI from 0.120–0.140, and zeta potential from –28.20 to −23.00 mV. Moreover, the drug-loaded exosomes displayed sustained drug release profile at pH 7.4. Further, on functionalization by folic acid, the drug-loaded functionalized exosomes resulted in higher cellular uptake both qualitatively and quantitatively, significant inhibition of cell growth as expressed by IC 50 values and increased apoptotic index. Moreover, the FA functionalized drug-loaded exosomes showed significant inhibition in cell migration compared to those without functionalization. Although the data in hand is quite impressive, additional in-vivo studies are required to prove the synergistic efficacy of the exosomal formulation.

## Figures and Tables

**Table 1:** Particle size, PDI, Zeta potential, Entrapment efficiency, and Drug loading of different exosomal formulations.

Samples	Size (nm) *	PDI *	Zeta Potential (mV) *	Entrapment Efficiency (EE%) *	Drug Loading *
**Exosomes**	83.5 ± 2.6	0.145 ± 0.08	−23.7 ± 0.9	-	-
**Exo-PAC**	86.6 ± 2.6	0.156 ± 0.019	−25 ± 0.5	85 ± 3.8	28.3 ± 3.9
**Exo-5-FU**	88.2 ± 2.8	0.200 ± 0.012	−28.2 ± 1.3	78 ± 2.2	19 ± 4.6
**FA-Exo-PAC**	95.3 ± 3.4	0.158 ± 0.016	−27.09 ± 1.6	82 ± 5.6	26.2 ± 2.6
**FA-Exo-5-FU**	97.3 ± 3.4	0.186 ± 0.022	−26.2 ± 1.5	75 ± 2.5	17 ± 2.9

Note: Exo-PAC-Paclitaxel loaded exosomes, Exo-5-FU-5-Fluorouracil loaded exosomes, FA-Exo-Pac and FA-Exo-5-FU: Folic acid conjugated paclitaxel or 5-Fluorouracil loaded exosomes, * Values represents the mean of three replicate with standard deviation (Mean ± SD).

**Table 2:** Size and drug content of exosomes and drug-loaded exosomes, before and after freeze-drying.

Freeze-Drying	Before Freeze-Drying			After Freeze-Drying (5% Trehalose)		
	Size (nm)	PDI	Drug Content	Size (nm)	PDI	Drug Content
**Exo**	83.5 ± 2.6	0.145 ± 0.08	-	84.2 ± 3.1	0.148 ± 0.06	-
**Exo-PAC**	86.6 ± 2.6	0.156 ± 0.019	28.3 ± 3.9	87.2 ± 2.2	0.161 ± 0.015	28.2 ± 2.5
**Exo-5-FU**	88.2 ± 2.8	0.200 ± 0.012	19 ± 4.6	88.9 ± 2.6	0.198 ± 0.018	18.9 ± 3.5
**FA-Exo-PAC**	95.3 ± 3.4	0.158 ± 0.06	26.2 ± 2.6	94.3 ± 2.4	0.158 ± 0.016	26.1 ± 2.8
**FA-Exo-5-FU**	97 ± 3.4	0.186 ± 0.022	17 ± 2.9	96.9 ± 1.8	0.186 ± 0.021	16.9 ± 2.6

**Table 3:** Cytotoxic effect of PAC, 5-FU, and their formulations in MCF-7 and MDA-MB-231 cell lines at 24, 48, and 72 h.

Group	IC_50_ (µM)					
	24 h		48 h		72 h	
	MCF-7	MDA-MB-231	MCF-7	MDA-MB-231	MCF-7	MDA-MB-231
Exo *	15.1 ± 2.2	18.3 ± 1.8	13 ± 1.6	15.8 ± 2.1	15.2 ± 1.4	17.8 ± 2.2
**Free PAC**	0.8 ± 0.2	1.1 ± 0.1	0.6 ± 0.5	0.9 ± 0.2	0.78 ± 0.2	1.2 ± 0.1
**Free 5-FU**	3.8 ± 0.2	15.6 ± 1.9	2.9 ± 0.5	14.6 ± 0.8	3.6 ± 0.3	14.8 ± 2.2
**Free-PAC/5-FU**	0.6 ± 0.3	0.9 ± 0.4	0.52 ± 0.11	0.78 ± 0.1	0.69 ± 0.21	0.96 ± 0.3
**Exo-PAC**	0.63 ± 0.28	0.88 ± 0.2	0.54 ± 0.1	0.76 ± 0.21	0.67 ± 0.1	0.9 ± 0.22
**Exo-5-FU**	3.0 ± 0.21	12.2 ± 0.23	2.2 ± 0.4	10.9 ± 0.54	2.9 ± 0.31	11.8 ± 0.2
**Exo-PAC/5-FU**	0.4 ± 0.2	0.6 ± 0.12	0.28 ± 0.12	0.52 ± 0.3	0.48 ± 0.2	0.78 ± 0.23
**FA-EXO-PAC/5-FU**	0.2 ± 0.1	0.50 ± 0.22	0.11 ± 0.08	0.21 ± 0.22	0.18 ± 0.09	0.48 ± 0.1

* Exosomes concentration were represented in µg/mL and remaining data were represented in moles. Values are represented as mean ± SD (*n* = 4).

## Data Availability

Not applicable.
